# Microglial histone deacetylase-3 conditional deletion attenuates neurological deficits after intracerebral hemorrhage

**DOI:** 10.3389/fncel.2026.1734472

**Published:** 2026-02-24

**Authors:** Noah J. Watson, Hongyan Xu, Sangeetha Sukumari-Ramesh

**Affiliations:** Department of Pharmacology and Toxicology, Medical College of Georgia, Augusta University, Augusta, GA, United States

**Keywords:** epigenetic mechanisms, HDAC3, histone deacetylases, inflammation, intracerebral hemorrhage

## Abstract

Stimulation of the innate immune system after intracerebral hemorrhage (ICH), characterized by microglial activation, contributes to ICH-induced neuroinflammation and brain damage. Despite the efficacy of broad-spectrum histone deacetylase (HDAC) inhibitors in improving acute neurological outcomes after ICH, the isoform- or cell-specific roles of histone deacetylases (HDACs) after ICH remain largely understudied. Given the emerging role of HDAC3 in various neuropathological conditions, we herein evaluate the functional role of microglial HDAC3 after ICH using newly developed microglia-specific HDAC3 conditional knockout mice (cKO). The microglia-specific conditional deletion of HDAC3 in male and female mice improved acute and long-term neurobehavioral outcomes following ICH. Furthermore, conditional deletion of HDAC3 in microglia significantly attenuated the expression of proinflammatory mediators, such as *Nos2*, *S100A9*, *TNF*-*α*, and *IL-6*, and augmented the expression of anti-inflammatory mediators, such as *Arg-1*, in the ipsilateral brain region following ICH. This observation was found to be concomitant with a reduction in the number of Iba1-positive cells, further implicating attenuation of neuroinflammatory response after ICH. Moreover, conditional deletion of HDAC3 in microglia did not alter hematoma volume after ICH, suggesting that the observed effects are independent of hematoma size. Overall, the data implicate a novel role of microglial HDAC3 in regulating neurological deficits after ICH in male and female subjects.

## Introduction

Intracerebral hemorrhage (ICH) is the second most common subtype of stroke, characterized by the rupture of blood vessels within the brain, causing blood to flow into the surrounding neural tissue. With a 30-day mortality rate of 50% (Ariesen et al., 2003; Wasil and Lichtman, 2005; Qureshi et al., 2001), ICH is the most deadly type of stroke (An et al., 2017), and the survivors often exhibit a poor quality of life. Of the survivors, only about 20% regain functional independence (Elliott and Smith, 2010). The incidence of ICH is increasing worldwide and is expected to increase by two-fold by 2050, largely due to population aging and the widespread use of anticoagulants (Ariesen et al., 2003; Wasil and Lichtman, 2005; Qureshi et al., 2001; Bako et al., 2022). Despite recent advances in clinical trials, effective treatment options are limited for ICH, necessitating further investigation into the complex pathophysiology of the disease (Hatakeyama et al., 2013; Huang et al., 2002; Hamada and Matsuoka, 2000).

ICH often results in severe brain injury, which is typically categorized as primary and secondary brain damage. The primary brain injury results from vascular rupture and hematoma formation (Lok et al., 2011), causing neural tissue damage proximal to the hematoma and elevated intracranial pressure (Dasari et al., 2021). Multiple clinical trials have attempted to alleviate the primary damage associated with ICH through surgical intervention, but there has been no significant improvement in comparison to conservative management (Hanley et al., 2019; Mendelow et al., 2015; Mendelow et al., 2013). In contrast to primary damage, secondary brain injury is characterized by molecular and cellular responses to the initial brain insult (Lok et al., 2011). The prominent mechanisms of secondary injury are neuroinflammation and oxidative stress, which can culminate in acute and long-term neurological deficits (Lok et al., 2011).

Histone deacetylases (HDACs) regulate gene expression, including genes associated with inflammatory signaling, by removing acetyl groups from the histones (Zhang et al., 2017). There are several isoforms of HDACs classified into four HDAC classes (I–IV), and HDAC isoforms may have distinct functions in normal physiology and pathology (Graff and Tsai, 2013). Our lab has previously demonstrated the role of epigenetic mechanisms in the pathophysiology of ICH. Along these lines, inhibiting HDACs with broad-spectrum as well as Class I HDAC inhibitors improved acute neurological outcomes post-ICH in mice (Sukumari-Ramesh et al., 2016; Bonsack and Sukumari-Ramesh, 2021). However, the isoform-specific role of HDAC after ICH needs to be identified, as this could result in the development of efficacious treatment strategies. Along these lines, emerging studies indicate a critical role for a class I HDAC isoform, histone deacetylase 3 (HDAC3), in inflammation associated with diverse pathologies (Zhao et al., 2019; Kuboyama et al., 2017; Zhu et al., 2017). Moreover, microglia are key regulators of ICH-induced neuroinflammation and neurological deficits (Colonna and Butovsky, 2017). Based on these findings, we hypothesized that microglia-specific conditional deletion of HDAC3 would attenuate the neuroinflammatory response and improve outcomes post-ICH. To test the hypothesis, we employed a genetic approach and developed a mouse model of microglia-specific conditional HDAC3 knockout using Cre-Lox technology. Given that ICH incidence is increasing in both sexes, the present study was conducted in both male and female mice.

## Materials and methods

### Mice

The animal studies were reviewed and approved by the Institutional Animal Care and Use Committee, and the studies were conducted per the Guide for the Care and Use of Laboratory Animals and the ARRIVE guidelines for reporting animal experiments. Mice were housed in a pathogen-free setting at a 12-h light/12-h dark cycle. Food and water were provided ad libitum.

To generate microglia-specific conditional HDAC3 knockout mice, HDAC3 floxed mice, with LoxP sites on either side of HDAC3 exon 7 [HDAC3^fl/fl^; originally generated by Dr. Scott W. Hiebert lab (Bhaskara et al., 2008) and acquired from Dr. Meghan E McGee-Lawrence’s lab at Augusta University] were bred with Cx3cr1CreER or Cx3cr1 mice (Stock No: 021160; The Jackson Laboratory, United States). The genotypes of the offspring were confirmed by PCR, using genomic DNA extracted from mice tails with a kit from Qiagen (Hilden, Germany). The 3-week-old mice (both the conditional knock out and the experimental control) were administered Tamoxifen (TAM, 75 mg/kg i.p. per day; four alternate days). To verify genetic recombination in brain tissue, DNA was isolated from the mouse brain tissue using a Thermo Scientific GeneJET DNA purification kit, and PCR was carried out on the purified DNA using HDAC3 primers. The primer sequences used for PCR are provided in Supplementary Table 1.

For studies, mice were randomly assigned. The power analysis (PASS software, NCSS LLC) was conducted using previous data that implied *n* = 6 for RT-PCR to obtain a power of 80%. The study employed 172 mice in total. Of those, nine mice were excluded. The exclusion criteria included the absence of a hematoma or animal death. The data from male and female subjects were separately analyzed to determine if there is a sex-dependent effect on the functional role of microglial HDAC3 after ICH.

### Induction of ICH

Male and female mice (8–12 weeks old) were anesthetized with an i.p. injection of ketamine and xylazine (100 mg/kg and 10 mg/kg, respectively). The mice were then secured on a stereotaxic head frame (Stoelting, WI, United States), and a small animal temperature controller maintained the body temperature at 37 ± 0.5 °C. For a cranial opening, a high-speed dental drill (Dremel, United States) was used, and the burr hole was made 2.2 mm lateral to the bregma. A 26-G Hamilton syringe containing 0.04 U of type IV bacterial collagenase (Sigma, St. Louis, MO, United States) in 0.5-μL saline was inserted 3.0 mm into the left striatum through the burr hole to induce spontaneous brain hemorrhage (Sukumari-Ramesh et al., 2012a, 2012b). For sham or experimental control, animals underwent the surgical procedure as detailed above, but 0.5 μL saline was injected into the brain striatum. Upon removal of the needle, the burr hole was sealed with bone wax, and the incision was closed. Mice were kept at 37 °C until recovery.

### Neurobehavioral analysis

The neurobehavioral outcome was determined using a 24-point scale (Wu et al., 2012; King et al., 2011). The scale comprises six individual behavioral tests: climbing, circling, compulsory circling, whisker response, bilateral grasp, and beam walking. Each test was scored from 0 (performs with no impairment) to 4 (severe impairment). The sum of the scores on all six tests, or the composite neurological deficit score, is a measure of neuroglial deficits (sensory-motor deficits). The neurobehavioral analysis was carried out independently by an investigator blinded to the experimental groups.

### RNA analysis

Mice were anesthetized deeply with isoflurane and perfused transcardially with ice-cold phosphate-buffered saline, pH 7.4 (PBS). Three millimeters brain sections containing the hematomal and perihematomal brain regions post-ICH were collected using a brain matrix and flash-frozen in liquid nitrogen. Brain tissues were then homogenized in 800 μL Trizol reagent (Invitrogen, Catalogue number: 15596018). The homogenate was then incubated for 5 min at room temperature before adding 160 μL chloroform. Samples were then centrifuged at 12,000 g for 15 min at 4 °C, and the aqueous phase was collected into fresh tubes. Four hundred microliters isopropanol was added to the aqueous phase, and the solution was incubated for 10 min at room temperature before centrifugation at 12,000 g for 10 min at 4 °C. The supernatant was decanted, and the remaining pellet was washed in 75% ethanol. Samples were then centrifuged at 7,500 g for 5 min. The supernatant was decanted, and the RNA pellets were air-dried for 10 min. Pellets were then resuspended in nuclease-free water and incubated in a heat block at 70 °C for 15 min. Quantitative RT-PCR was performed on a Bio-Rad C1000 Touch Thermal Cycler using a GoScript reverse transcriptase kit (Promega, Madison, WI) and SsoAdvanced Universal SYBR Green Supermix (BioRad, Hercules, CA). The RNA expression of *Nos2*, *S100A8*, *S100A9*, *IL-1β*, *IL-6*, *TNF*-*α*, *Arg-1*, and *CD206* was evaluated. Data were normalized to *GAPDH*, a housekeeping gene, and expressed as fold change compared with the control. The primer sequences used for the quantitative RT-PCR are provided as part of Supplementary Table 1.

### Immunohistochemistry

Deeply anesthetized mice were perfused transcardially with ice-cold PBS and brains were harvested from the animals and incubated overnight in 4% paraformaldehyde at 4 °C. Brains were then transferred to 30% sucrose at 4 °C until permeated. They were then cryopreserved with optimal cutting temperature (OCT) compound and sectioned at 20 μm using a cryostat. Briefly, sections were mounted on glass slides and treated with 10% normal donkey serum in PBS containing 0.4% Triton X-100 for 2 h. Sections were then incubated overnight at 4 °C with primary antibody [Iba1 (ionized calcium binding adaptor molecule), 1:100, goat polyclonal, Abcam, MA, United States; NeuN, 1:100, rabbit monoclonal, Cell Signaling Technologies, MA, United States; GFP, 1:100, mouse monoclonal, ThermoFisher, MA, United States]. Sections were washed 3 times with a 0.1% Triton X-100 in PBS solution and incubated with Alexa Fluor-tagged secondary antibody (Alexa Fluor 488 donkey anti-goat IgG, 1:1,000, Invitrogen, MA, United States; Alexa Fluor 594 donkey anti-goat IgG, 1:1,000, Invitrogen, MA, United States; Alexa Fluor 594 donkey anti-rabbit IgG, 1:1,000, Invitrogen, MA, United States; Alexa Fluor 488 donkey anti-mouse IgG, 1:1,000 Invitrogen, MA, United States) at room temperature in the dark for 1 h. The antibody catalogue numbers are provided in Supplementary Table 2. After washing, the sections were cover-slipped with a DAPI-containing mounting media (DAPI-Fluoromount-G; SouthernBiotech, AL, United States). Immunofluorescence was imaged using a Zeiss 780 inverted confocal laser microscope. As previously reported (Bonsack et al., 2016), we analyzed three nonconsecutive sections per animal and three fields around the hematoma. The number of immunoreactive cells per mouse was quantified using ImageJ software (NIH, United States), and averaged as positive cells per 0.1 mm^2^ in the peri-hematomal brain region (Bonsack et al., 2016).

### Fluoro-Jade B staining

Brain sections (20 μm) were re-hydrated and incubated with 0.06% KMnO4 for 15 min. Sections were then washed with distilled water, incubated for 30 min in a 0.001% Fluoro-Jade B solution, dried for 30 min at 37 °C, and cover-slipped using DPX mounting media. The sections were visualized with an excitation of 488 nm using a Zeiss 780 inverted confocal laser microscope. We used six sections per animal and examined different areas around the hematoma. Employing Image J software, the number of Fluro-Jade B-positive cells was quantified as detailed previously (Bonsack et al., 2017).

### Statistical analysis

The data analysis was performed using GraphPad Software, GraphPad Prism, or R software (version 4.5.1). The results are expressed as mean ± SD. The original/log-transformed data from each group were analyzed using the Shapiro–Wilk test to determine normal distribution. An unpaired *t*-test was used to analyze two groups that passed the normality test, and the Mann–Whitney test was used to analyze non-normal distributions. A one-way ANOVA followed by Tukey’s multiple comparisons test was used for four group comparisons. For neurobehavioral analysis, the Wilcoxon rank sum test (a nonparametric test) was performed for the two-group comparison at specific time points, and rank-based ANOVA (nonparametric test) was also used for longitudinal data analysis. A *p*-value <0.05 is considered statistically significant.

## Results

### Microglial histone deacetylase-3 conditional deletion improved acute neurological outcomes after ICH

**Figure 1:**
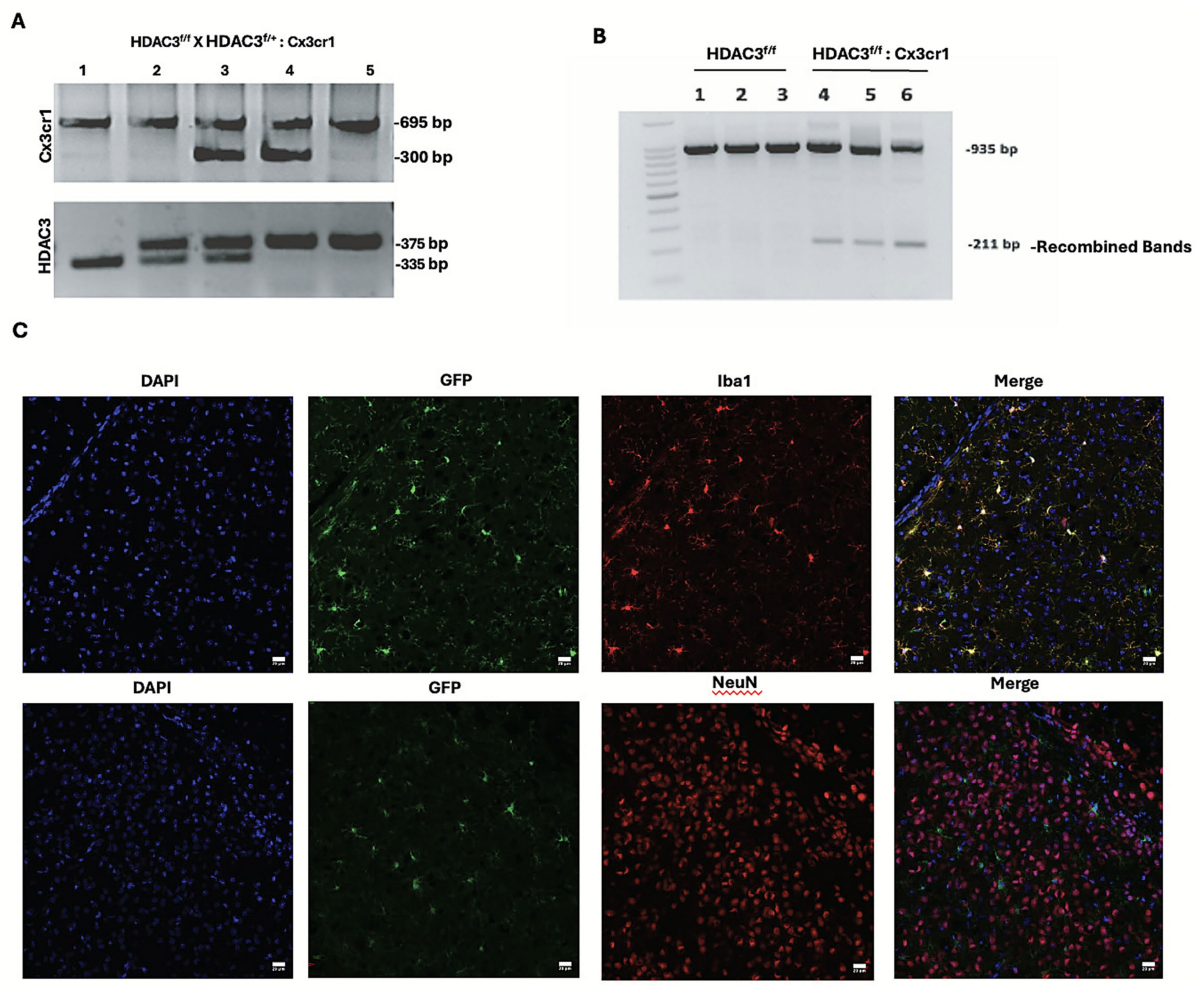
Validation of transgenic models: the cKO (HDAC3^f/f^: Cx3cr1Cre) and the experimental control (HDAC3^f/f^). **(A)** Lanes 1–5 represent the genotyping data for C57BL/6J (loading control), HDAC3^f/+^, HDAC3^f/+^: Cx3cr1Cre, HDAC3^f/f^: Cx3cr1Cre, and HDAC3^f/f^, respectively. The genotyping was performed as detailed in the Methods section, and the PCR products were separated by electrophoresis on an agarose gel. **(B)** Lanes 1–3 and lanes 4–6 indicate brain tissue recombination analysis results from the control and cKO mice, respectively. The figure depicts the pattern of floxed (935 bp) and null (211 bp) bands on an agarose gel after electrophoresis, suggesting that the recombination occurs in brain tissue derived from the cKO mice. **(C)** HDAC3^f/f^: Cx3cr1Cre mice brain sections were subjected to dual-label immunohistochemistry analysis as detailed in the Methods section. The figure (top panel) illustrates nearly exclusive GFP expression under the control of Cx3cr1 promoter in Iba1-positive cells, suggesting that the genetic recombination responsible for the conditional deletion occurs predominantly in Iba1-positive cells or microglia. The bottom panel illustrates that there was no expression of GFP in NeuN-positive neurons in the brain section derived from HDAC3^f/f^: Cx3cr1Cre mice (*n* = 3/group).

To determine the functional role of microglial HDAC3 after ICH, we first developed microglia-specific HDAC3 conditional knockout (cKO) mice. For that, Cx3cr1CreER mice were bred with HDAC3^f/f^ (HDAC3^flox/flox^) to develop HDAC3^f/+^: *Cx3cr1*, which was then bred with HDAC3^f/f^ to generate the conditional knockout or cKO (HDAC3^f/f^: *Cx3cr1*). The breeding strategy enabled the generation of HDAC3^f/f^ (experimental control) and HDAC3^f/f^: *Cx3cr1* (cKO) in the same litter (Figure 1A). The two groups (experimental control and cKO) were then administered Tamoxifen as detailed in the Methods section. TAM injections were followed by a minimum 4-week waiting period before stereotactic surgery, allowing the renewal of the Cx3cr1-positive monocytes/macrophages with wild-type cells, whereas microglia maintain the gene deletion (Gao et al., 2017). Additionally, the genetic recombination assay confirmed the recombination in the brain tissue of the cKO mice (Figure 1B). Also, Cx3cr1CreER offers a unique experimental system that explicitly targets microglia with high efficiency (Goldmann et al., 2013) and expresses an enhanced yellow fluorescent protein (YFP) under the control of the Cx3cr1 promoter (Ye et al., 2019). Our studies demonstrate GFP (green fluorescent protein, a mutant of YFP that can be recognized by GFP antibody) expression predominantly in Iba1-positive cells in the mouse brain of the cKO (Figure 1C). The data suggest that Cre recombinase, the enzyme responsible for the genetic recombination, localizes predominantly to microglia. Also, GFP expression was absent in NeuN-positive neurons in the cKO mice (Figure 1C). Together, the data validate the conditional knockout model.

**Figure 2:**
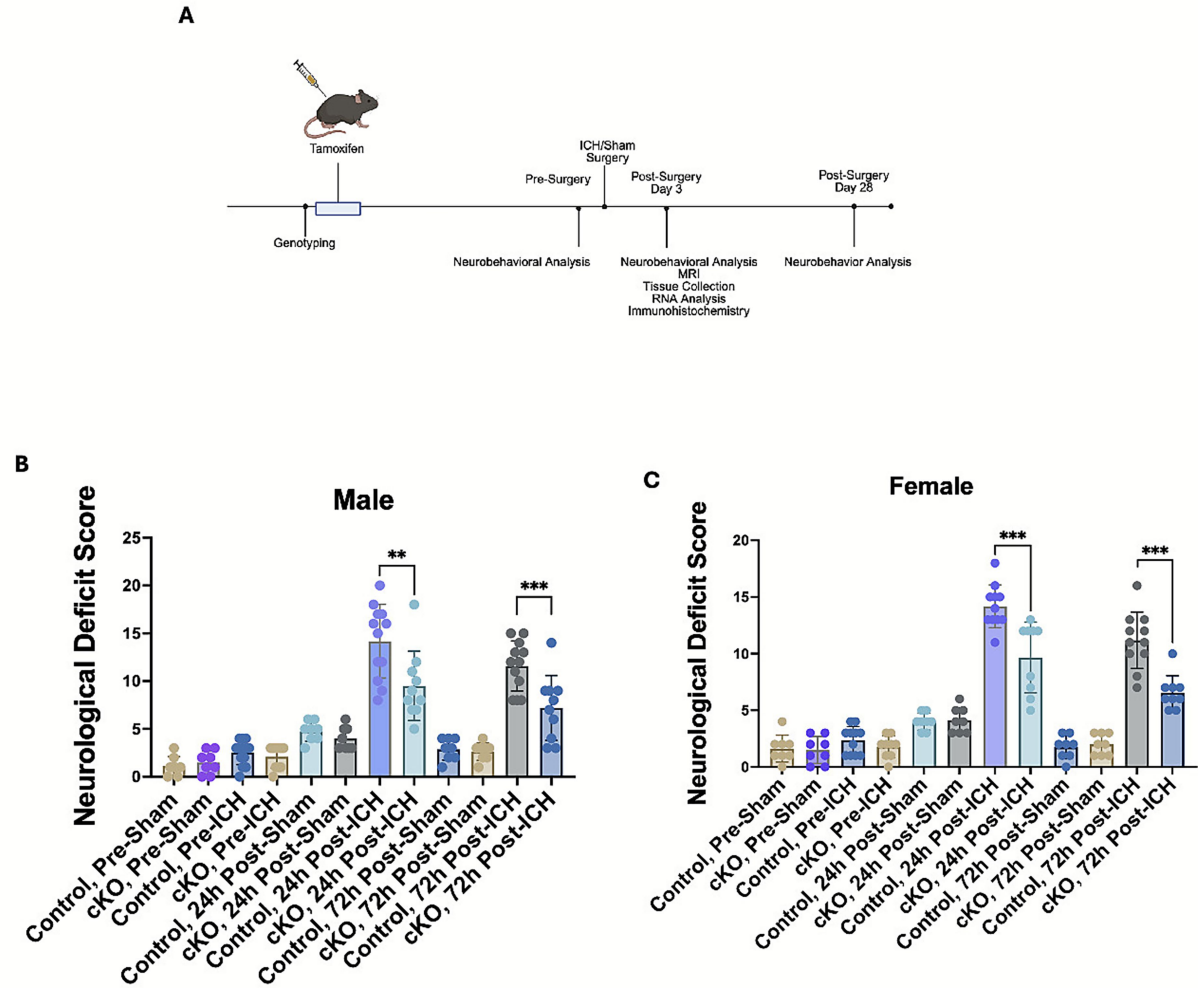
Microglial histone deacetylase-3 conditional deletion improved acute neurological outcomes after ICH. **(A)** Illustrates the experimental timeline. The male cKO **(B)** and female cKO **(C)** exhibited reduced composite neurological deficit scores on day 1 and day 3 post-ICH compared to the respective control. Mice were subjected to neurobehavioral analysis per the Methods section, and a composite neurological deficit score was calculated as the sum of scores across all six tests, with a maximum neurological deficit score of 24. The data (provided in Supplementary Table 3) were analyzed using a nonparametric test (Wilcoxon rank-sum test) with R software (version 4.5.1). The figure shows group comparisons at specific time points, with results expressed as mean ± SD (^**^p < 0.05 and ^***^p < 0.01) (*n* = 8–13/group).

To evaluate the functional role of HDAC3, the male and female cKO and control mice were subjected to ICH or sham, and neurobehavioral analysis at day 1 and day 3 post-surgery was performed as detailed in the methods section. Neurobehavioral analysis was also performed on animals a day prior to the surgery. Figures 2A illustrates the experimental timeline. Irrespective of sex, no difference in neurobehavior was observed between the experimental control and the cKO group before surgery, indicating that the genetic deletion of HDAC3 in microglia did not confer any inherent neurobehavioral changes (Figures 2B,C). Notably, at day 1 and day 3 post-ICH, a significant reduction in neurological deficits was observed in male and female cKO compared to the respective control, implicating a novel role of microglial HDAC3 in regulating neurological outcomes after ICH (Figures 2B,C).

### Microglial histone deacetylase-3 conditional deletion attenuated microglial activation post-ICH

**Figure 3:**
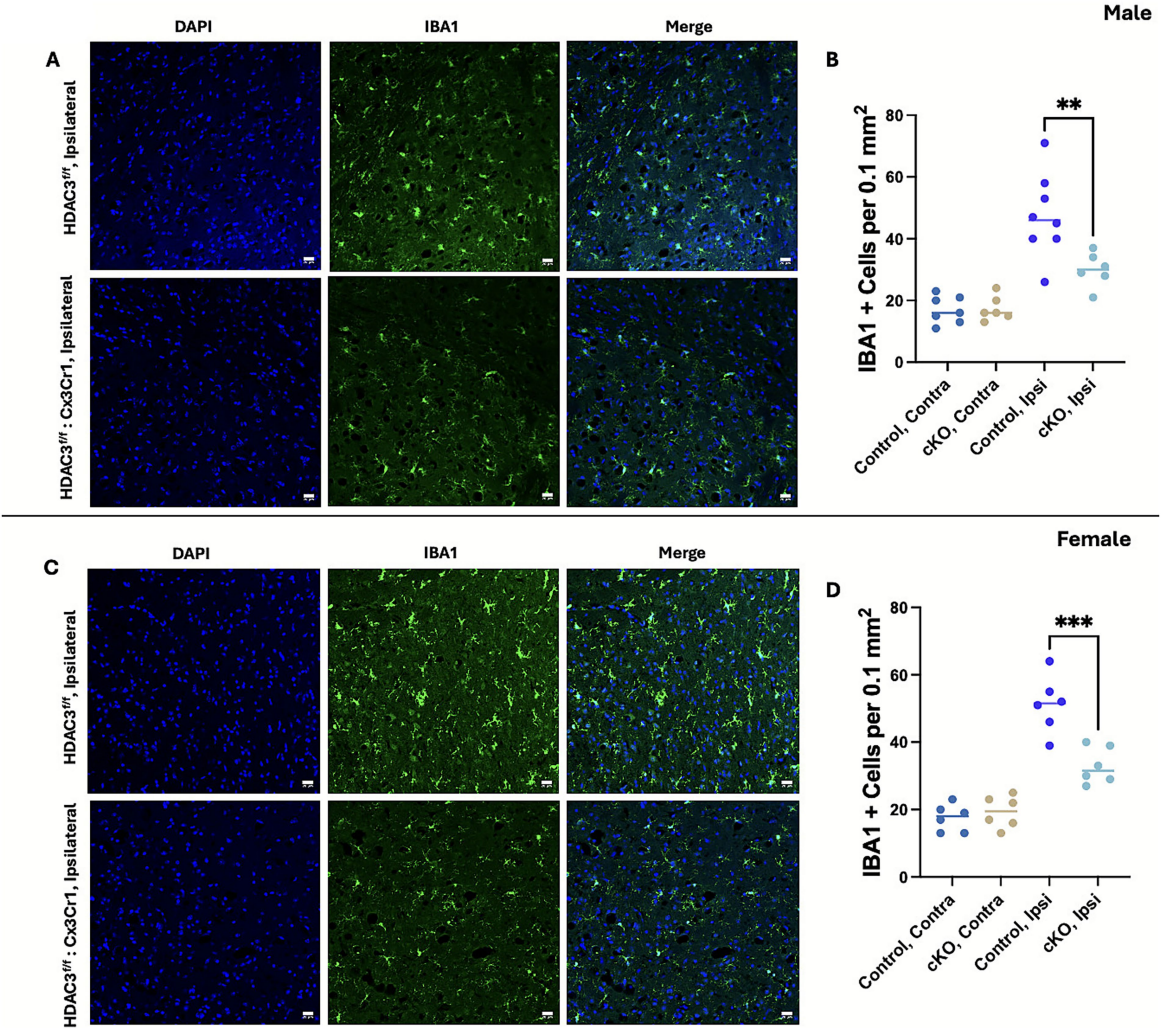
Microglial histone deacetylase-3 conditional deletion attenuated the number of Iba1-positive cells in the ipsilateral brain region, a measure of microglial activation, post-ICH. Mice brain sections were subjected to Iba1 immunohistochemical analysis as detailed in the Methods section. The sections were counterstained with DAPI and subjected to confocal analysis. The figure illustrates representative Iba1 staining and the quantification of Iba1 + cells in the ipsilateral peri-hematomal region of male cKO **(A,B)** and female cKO **(C,D)** compared to the contralateral brain region of the respective control at day 3 post-ICH. A one-way ANOVA followed by Tukey’s multiple comparisons test was used to analyze the data (details of the analysis results are provided in Supplementary Table 4). The results are expressed as mean ± SD (^**^p < 0.01 and ^***^p < 0.001) (*n* = 6-8/group).

Next, we evaluated whether HDAC3 conditional deletion modulated microglial activation after ICH. Brain injury-induced microglial activation is characterized by an increase in microglial number and enhanced cellular hypertrophy in the ipsilateral brain region. Profound microglia activation, a prominent feature of neuroinflammation, occurs at day 3 post-ICH (Bonsack et al., 2016), and microglial activation was determined using immunohistochemical analysis using a marker of microglia/macrophages, Iba1. The number of Iba1-positive cells was significantly reduced in the ipsilateral perihematomal brain regions of both male and female cKO at day 3 post-ICH compared to the respective control (Figures 3A–D). Also, Iba-1-positive cells exhibited a less activated phenotype (less hypertrophic) in the male and female cKO compared to the respective control. Together, the data suggest that microglial HDAC3 conditional deletion could reduce ICH-induced neuroinflammatory response, which, in turn, could culminate in improved outcomes.

### Microglial histone deacetylase-3 conditional deletion attenuated neurodegeneration in females but not in males post-ICH

**Figure 4:**
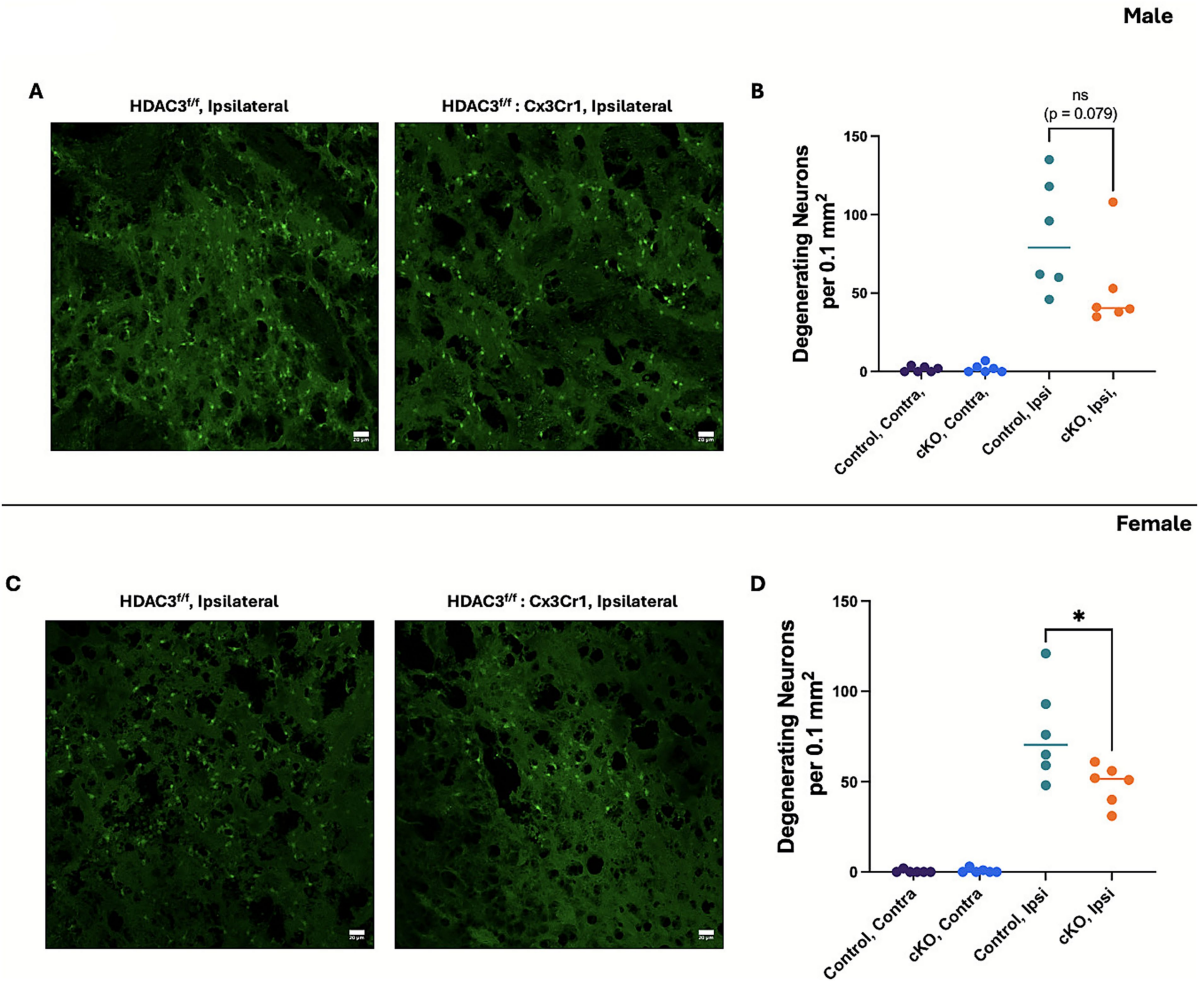
Microglial histone deacetylase-3 conditional deletion attenuated neurodegeneration in females but not in males post-ICH. Mice brain sections were subjected to Fluoro-Jade B staining as per the Methods section. The figure illustrates representative Fluoro-Jade B staining, a measure of neurodegeneration, and the quantification of Fluoro-Jade B-positive cells in the ipsilateral peri-hematomal region of the male cKO **(A,B)** and female cKO **(C,D)** compared to the contralateral brain region of the respective control at day 3 post-ICH. A one-way ANOVA followed by Tukey’s multiple comparisons test was used to analyze the data (details of the analysis results are provided in Supplementary Table 5). The results are expressed as mean ± SD (^*^p < 0.05) (*n* = 6/group).

To assess neurodegeneration, the brain sections from the male and female control and cKO mice at day 3 post-ICH were subjected to Fluoro-Jade B staining. In the male cKO, the number of Fluoro-Jade B-positive cells exhibited a downward trend in comparison to the respective control; however, the reduction did not reach statistical significance (Figures 4A,B). By contrast, in the female cKO mice, there was a statistically significant reduction in the number of Fluoro-Jade B-positive cells (Figures 4C,D), suggesting that HDAC3 deletion in microglia attenuates neurodegeneration after ICH in female subjects. Together, the data suggest that there could be sex-based differences in HDAC3-mediated regulation of neurodegeneration after ICH, warranting further investigation.

### Microglial histone deacetylase-3 conditional deletion modulated inflammatory gene expression in the brain after ICH

**Figure 5:**
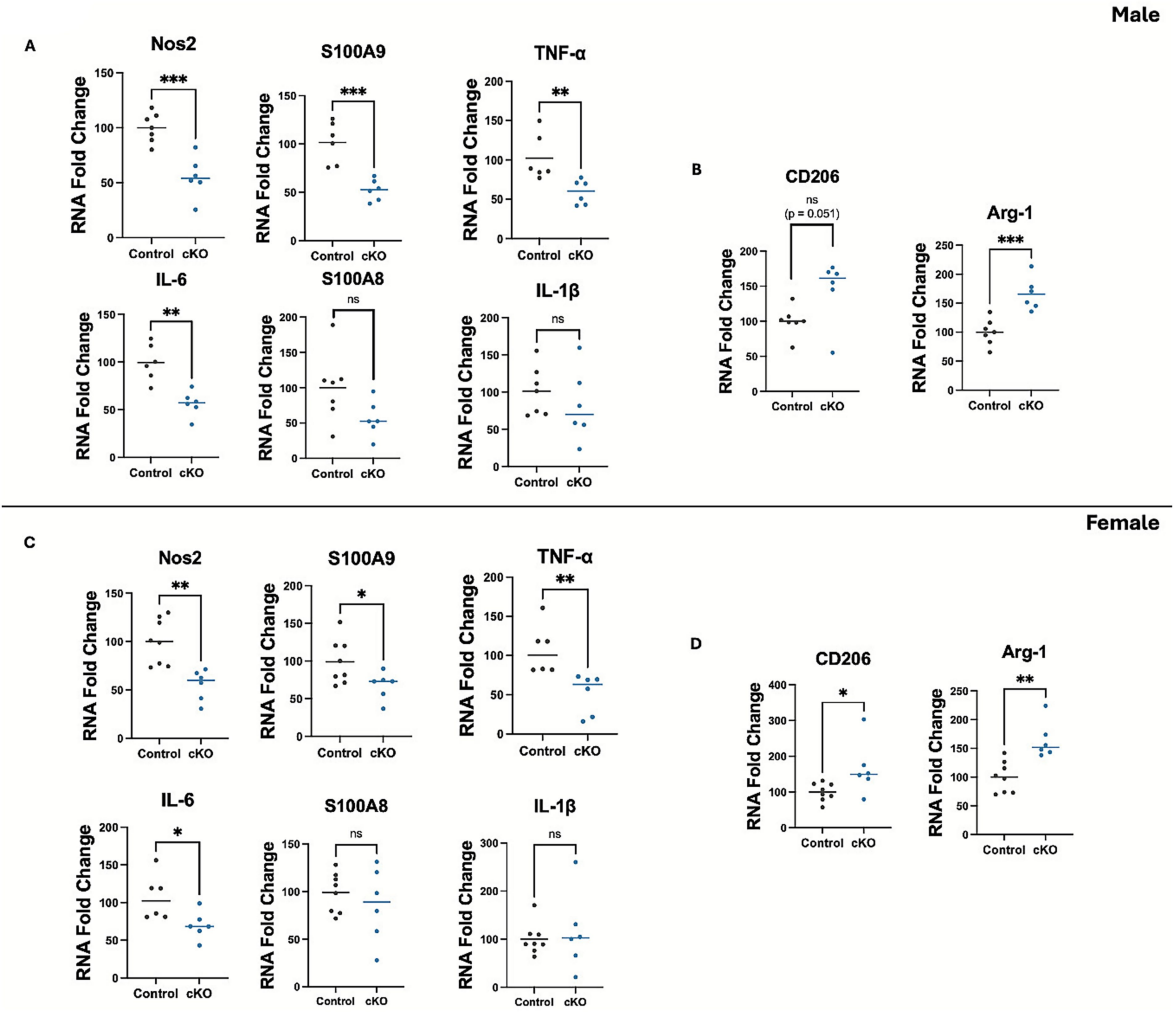
Microglial histone deacetylase-3 conditional deletion modulated pro/anti-inflammatory mediator expression in the brain after ICH. The male **(A,B)** and female **(C,D)** cKO exhibited reduced expression of several proinflammatory mediators and enhanced expression of anti-inflammatory mediators after ICH. Briefly, the ipsilateral brain region was subjected to RNA analysis, as detailed in the Methods. Data were normalized to *GAPDH*. An unpaired *t*-test was used to analyze two groups that passed the normality test, and the Mann–Whitney test was used to analyze non-normal distributions (details of the analysis results are provided in Supplementary Table 6). Data are expressed as fold change compared with the control and expressed as mean ± SD (^*^p < 0.05, ^**^p < 0.01, and ^***^p < 0.001) (*n* = 6/group).

**Figure 6:**
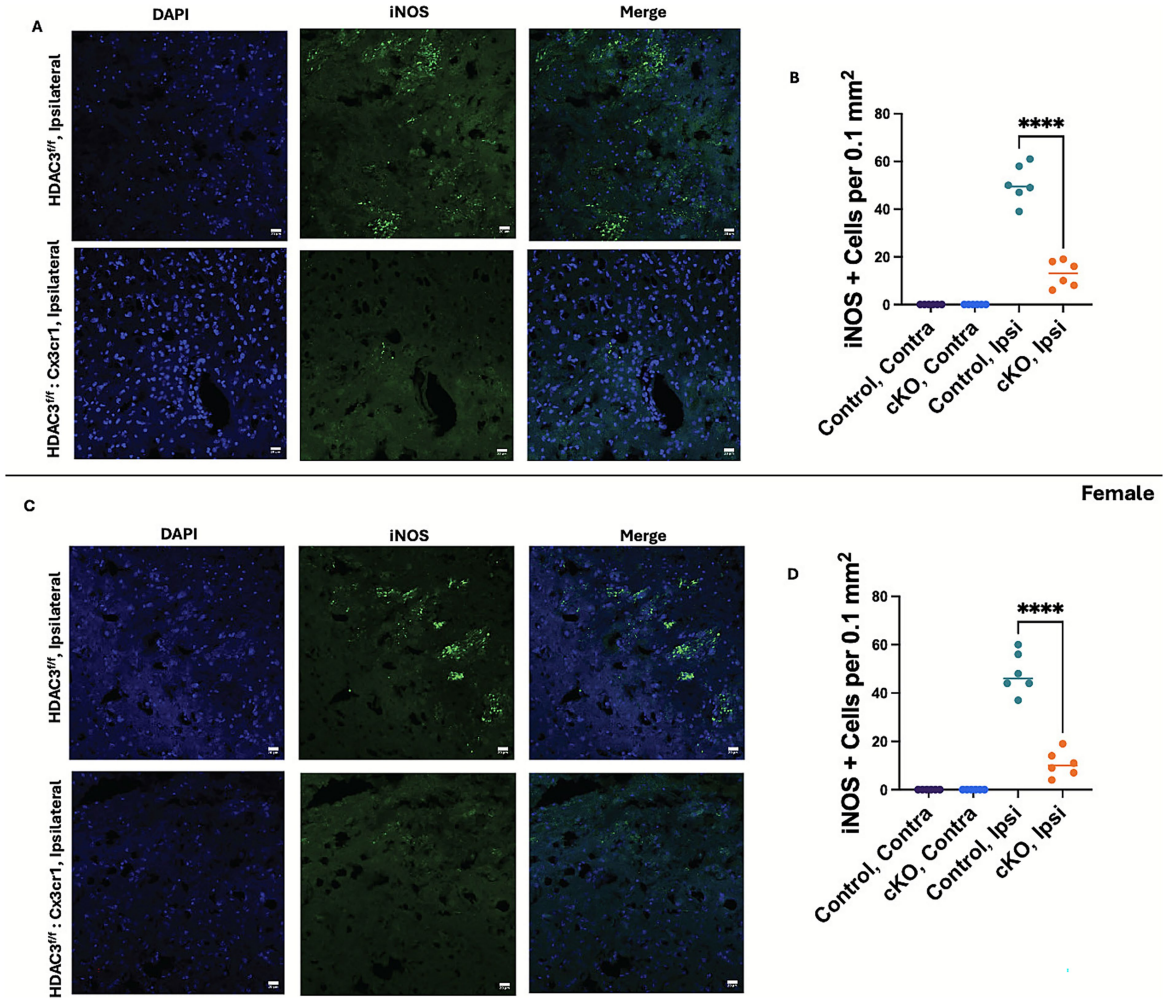
Microglial histone deacetylase-3 conditional deletion attenuated iNOS expression in the brain after ICH. Mice brain sections were subjected to iNOS immunohistochemical analysis as detailed in the Methods. Subsequently, the sections were counterstained with DAPI and subjected to confocal analysis. The figure illustrates representative iNOS immunostaining and the quantification of iNOS-positive cells in the peri-hematomal region of the male cKO **(A,B)** and female cKO **(C,D)** compared to the contralateral brain region of the respective control at day 3 post-ICH. A one-way ANOVA followed by Tukey’s multiple comparisons test was used to analyze the data (details of the analysis results are provided in Supplementary Table 7). The results are expressed as mean ± SD (^****^p < 0.0001) (*n* = 6/group).

To further examine the role of HDAC3 in the neuroinflammatory response after ICH, the RNA expression levels of proinflammatory mediators were assessed at day 3 post-ICH. HDAC3 conditional deletion in both male and female subjects significantly reduced the levels of proinflammatory mediators, such as *Nos2*, *S100A9*, *TNF-α*, and *IL-6* after ICH (Figures 5A,C) compared to the respective control. Interestingly, there was no change in the expression of *S100A8* and *IL-1β* (Figures 5A,C), suggesting HDAC3 may differentially regulate proinflammatory mediators after ICH. Furthermore, we found that the anti-inflammatory marker CD206, which is often expressed in a subset of microglia/macrophages, was upregulated in the female cKO compared to the control after ICH (Figure 5D). However, despite an upward trend, there was no significant increase in *CD206* expression in male cKO mice compared to the respective controls, implicating a sex-based difference in HDAC3-mediated regulation of *CD206* expression after ICH. Intriguingly, in both male and female cKO mice, another anti-inflammatory microglia or macrophage marker, *Arginase-1(Arg-1)*, was significantly upregulated after ICH compared to the respective control (Figures 5B,D). This observation is critical as there was also a concomitant reduction in the protein (Figures 6A–D) and RNA expression of the proinflammatory microglia/macrophage marker iNOS in the cKO group compared to the respective control. Together, the data suggest that microglial HDAC3 could regulate microglial phenotypes, steering them towards a reparative phenotype after ICH.

### Microglial histone deacetylase-3 conditional deletion did not alter hematoma volume after ICH

**Figure 7:**
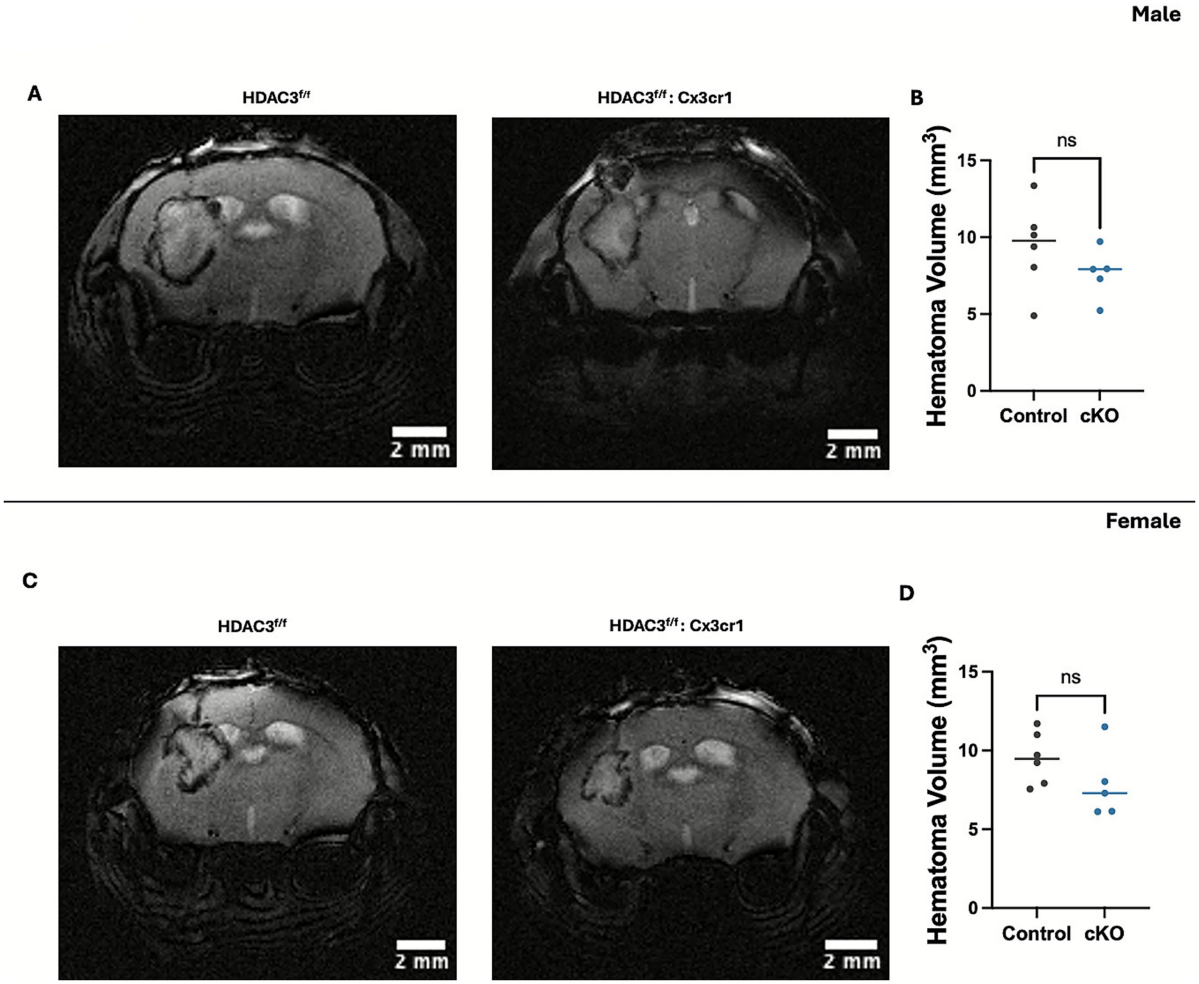
Microglial histone deacetylase-3 conditional deletion did not alter hematoma volume after ICH. Mice were subjected to SW-MRI, and the data were analyzed as per the Methods section. The figure illustrates SW-MRI depicting hematoma and its quantification in male cKO **(A,B)** and female cKO **(C,D)** compared to the respective control at day 3 post-ICH. The results are expressed as mean ± SD. An unpaired *t*-test was used for the analysis of data, and there was no significant difference (ns) between the two groups, as indicated [**(B)** *t* = 1.250; *p* = 0.2428 **(D)** *t* = 1.463; *p* = 0.1776] (*n* = 5–6/group).

Given the emerging role of HDAC3 in myeloid phagocytosis (Yang et al., 2024; Yang et al., 2017), which in turn could regulate hematoma size, we next questioned whether microglial HDAC3 regulates hematoma volume post-ICH. To determine this, the male and female cKO and control mice were subjected to ICH, and at day 3 post-ICH, susceptibility-weighted MRI (SW-MRI) was performed (Figures 7A–D). There was no significant change in hematoma volume in either male or female cKO group compared to the respective control. The data suggest that HDAC3 conditional deletion-mediated improvement in neurological outcome after ICH is independent of hematoma size.

### Microglial histone deacetylase-3 conditional deletion improved long-term functional outcomes after ICH

**Figure 8:**
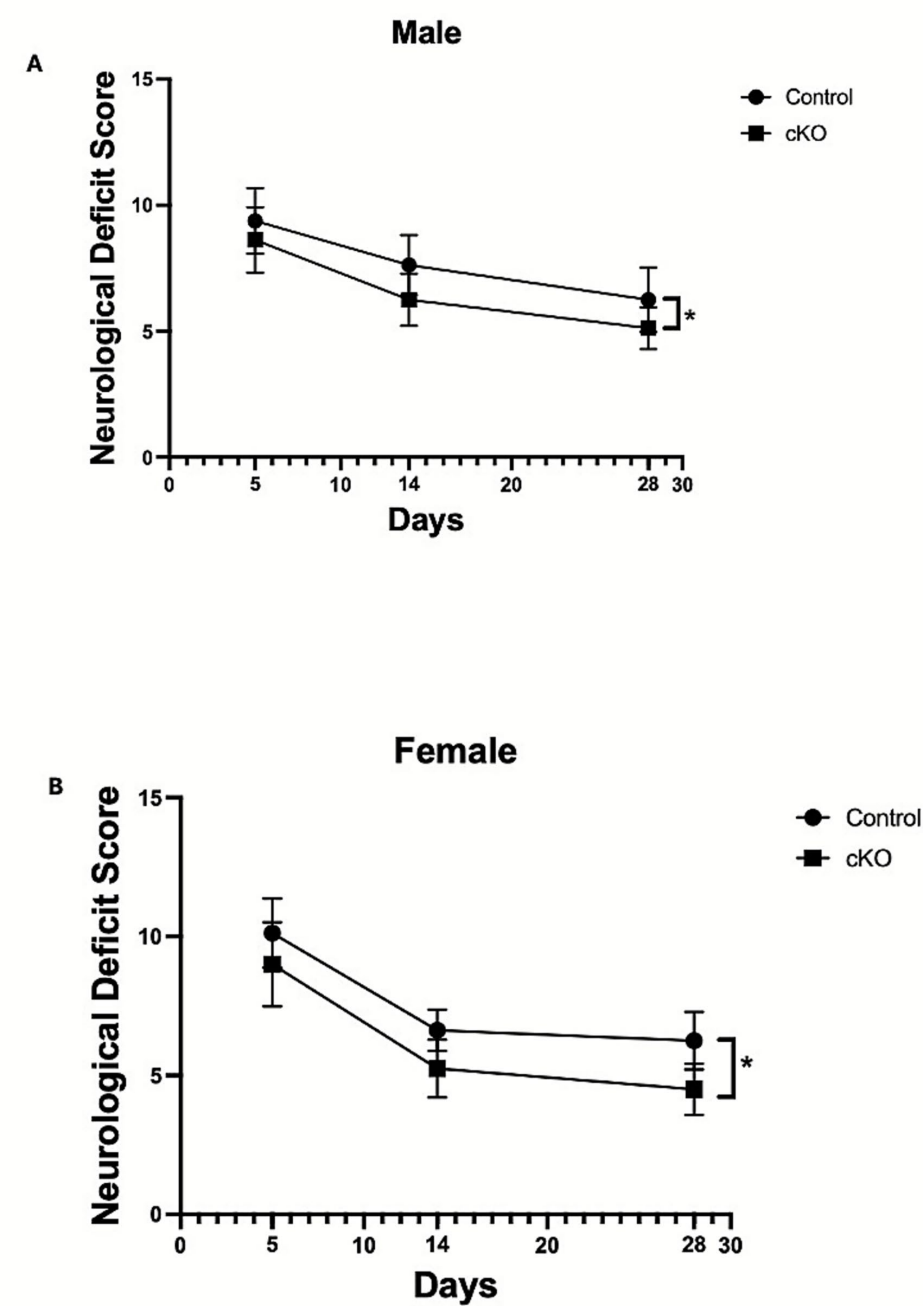
Microglial histone deacetylase-3 conditional deletion improved long-term functional outcomes after ICH. The male cKO **(A)** and female cKO **(B)** exhibited reduced long-term composite neurological deficit scores. Mice were subjected to neurobehavioral analysis per the Methods section, and a composite neurological deficit score was calculated as the sum of scores across all six tests, with a maximum neurological deficit score of 24. The data (provided in Supplementary Table 8) were analyzed using a rank-based ANOVA, a nonparametric test, with R software (version 4.5.1). The longitudinal data results are expressed as mean ± SD (^*^p < 0.05) (*n* = 8/group).

To evaluate the efficacy of microglial HDAC3 deletion in long-term functional recovery, the male and female cKO and control animals were subjected to ICH, and neurobehavioral analysis was performed at days 5, 14, and 28 post-ICH. Both male and female cKO mice exhibited improved long-term outcomes compared to the respective control (Figures 8A,B).

## Discussion

Despite the role of epigenetic mechanisms in the pathophysiology of ICH, the cell-specific or isoform-specific roles of HDACs after ICH remain largely understudied. The present study documents, for the first time, that microglia-specific conditional deletion of HDAC3 attenuates the ipsilateral brain expression of pro-inflammatory mediators and neurological deficits after ICH. Moreover, though the incidence of ICH is increasing in both males and females, very few preclinical studies to date have incorporated female subjects into their experimental design. As per the present study, conditional HDAC3 deletion-mediated improvement in outcomes was observed in both male and female mice following ICH. Together, the data suggest that targeting HDAC3 could be a promising therapeutic strategy for ICH, a significant public health concern with no effective treatment.

ICH encompasses a complex pathophysiology, which includes epigenetic changes in the brain. It has also been reported that HDAC inhibitors are effective in improving neurological outcomes after ICH. Along these lines, post-injury administration of SAHA, a pan-HDAC inhibitor, and entinostat, a class I HDAC inhibitor, improved acute neurological outcomes after ICH (Sukumari-Ramesh et al., 2016). In the studies, the improvement in neurological outcomes was concomitant with a reduction in neuroinflammation, a key regulator of brain damage after ICH. Also, another broad-spectrum or pan HDAC inhibitor, valproic acid (VPA), conferred neuroprotection after ICH with a reduction in the number of OX42 (CD11b, microglia/macrophage marker) -positive cells (Sinn et al., 2007), further emphasizing that HDACs can regulate neuroinflammation. Besides, in line with the role of epigenetic mechanisms, particularly histone acetylation in the pathophysiology of ICH, it was previously demonstrated that ICH results in altered HDAC activity in the brain, and a pan HDAC inhibitor, sodium butyrate, can improve cognitive function after ICH in rats (Okamura et al., 2023; Maejima et al., 2023). Moreover, another pan-HDAC inhibitor, scriptaid, was effective in reducing brain edema at day 3 post-ICH, hematoma volume at 7 days post-ICH, and white matter injury at 35 days post-ICH in mice (Yang et al., 2018). However, despite the efficacy of HDAC inhibition after ICH, broad-spectrum or pan HDAC inhibitors inhibit several isoforms of HDACs, so it is unclear whether inhibition of multiple isoforms or a specific isoform is responsible for the beneficial effects. Therefore, studies elucidating the isoform-specific and cell-specific roles of HDACs after ICH are required to further define the complex pathophysiology of ICH and develop efficacious treatment regimens. Also, isoform-specific HDAC inhibition may help reduce the side effects often associated with pan-HDAC inhibitors (Falkenberg and Johnstone, 2014; Liao, 2015).

Activated microglia are critical drivers of proinflammatory response by releasing detrimental proinflammatory mediators after ICH. Notably, microglial activation contributes to ICH-induced brain injury and loss of neurological function after ICH (Wang and Dore, 2007; Wang, 2010; Carmichael et al., 2008; Babu et al., 2012; Keep et al., 2012; Carson et al., 2006; Madangarli et al., 2019; Aronowski and Hall, 2005). Reduced microglial activation in the acute or subacute phase of ICH is often associated with improved functional outcomes. Furthermore, depletion of microglia conferred neuroprotection on day 3 post-ICH (Li et al., 2017), suggesting a detrimental role of microglia in the acute/subacute phase of ICH. Intriguingly, histone acetylation has been implicated in microglia/macrophage response to environmental cues (Halili et al., 2009; Shakespear et al., 2011; Roger et al., 2011; Cheray and Joseph, 2018), and several neuropathological conditions are associated with disrupted acetylation homeostasis (Rouaux et al., 2003; Boutillier et al., 2003). Herein, we report that the conditional deletion of HDAC3 in microglia attenuated both acute and long-term neurological deficits after ICH. Moreover, acute improvement in neurological outcomes after ICH was associated with a prominent reduction in microglial activation and the brain expression of various proinflammatory mediators. Reduction in microglial/ macrophage activation could be due to HDAC3-mediated regulation of the expression of proinflammatory mediators after ICH, which can regulate the activation of microglia/macrophages. Notably, per the present study, the brain expression of the proinflammatory microglial or macrophage marker iNOS/*Nos2* was significantly downregulated, and the anti-inflammatory microglial or macrophage marker *arginase-1* was significantly upregulated in male and female cKO mice after ICH, suggesting that HDAC3 conditional deletion can also shift the microglial activation phenotypes, thereby improving outcomes. This observation is supported by other studies implicating the regulation of *Nos2*/*Arg-1* dynamics in regulating inflammation resolution and microglial function (Meleady et al., 2023; Zhang et al., 2024). Intriguingly, iNOS and arginase-1 both compete for the same substrate, arginine (Rath et al., 2014), and microglial HDAC3 inhibition may confer neuroprotection after ICH by enhancing arginine catabolism by arginase-1, and thereby reducing the level of the neurotoxic catabolite of iNOS and driving anti-inflammatory microglial activation/response, improving outcomes. Of note, reducing proinflammatory microglial responses and concomitantly increasing anti-inflammatory microglial responses is regarded as an effective strategy for improving outcomes after ICH. These observations are consistent with the previous studies in which the specific deletion of HDAC3 in microglia attenuated brain inflammation after traumatic brain injury (Zhao et al., 2022) and ischemic stroke (Zhang et al., 2024; Liao et al., 2020). Besides its role in neuroinflammation, microglial deletion of HDAC3 has been demonstrated to increase the recruitment of brain-infiltrating macrophages and the clearance of debris after ischemic stroke (Li et al., 2025). Pharmacological inhibition of HDAC3 improved neurological outcomes in various neuropathological conditions (Rosete and Ciernia, 2024), implicating the potential of targeting HDAC3.

Despite the significance of the observations presented herein, studies have yet to be conducted to determine whether the observed changes in the expression of inflammatory mediators after ICH occur in microglia. Importantly, any change in microglial inflammatory response could also affect neuronal and non-neuronal cells, contributing to the overall brain response to injury after ICH and requiring further investigation. An acknowledged limitation of this study is that while we demonstrate GFP expression in Iba1-positive cells, or microglia, in the conditional knockout, indicating genetic recombination, the knockdown of HDAC3 in microglia has not been demonstrated using other biochemical methods due to the poor quality of HDAC3 immunostaining and the challenges with the isolation of microglia from the adult mouse brain. Importantly, though most of the data presented herein were in alignment between male and female cKO mice, HDAC3 conditional deletion significantly attenuated neurodegeneration in female subjects but not in males, which could be due to enhanced estrogen-conferred neuroprotection in females compared to males, and warrants further investigation.

Taken together, the data herein implicates a novel role of microglial HDAC3 in regulating neuroinflammation and neurological outcomes after ICH. Based on the present study, selective inhibition of HDAC3 using pharmacological agents should be evaluated to determine its efficacy in attenuating neurological deficits after ICH. Also, future studies are warranted to determine the precise mechanism by which microglial HDAC3 regulates neurological outcomes after ICH.

## Data Availability

The raw data supporting the conclusions of this article will be made available by the authors, without undue reservation.
